# From microgravity to hypergravity: Perspectives on circulatory adaptation through multiscale modelling

**DOI:** 10.1113/JP289417

**Published:** 2025-07-01

**Authors:** Dario Collia, Luigino Zovatto, Gianni Pedrizzetti

**Affiliations:** ^1^ Department of Cardiovascular Surgery Icahn School of Medicine at Mount Sinai New York NY USA; ^2^ BioMedical Engineering and Imaging Institute Icahn School of Medicine at Mount Sinai New York NY USA; ^3^ Department of Engineering and Architecture University of Trieste Trieste Italy

**Keywords:** cardiac haemodynamics, cardiovascular function, cardiovascular modelling, hyper‐gravity, micro‐gravity, space medicine

Gravity is a fundamental mechanical force on Earth, shaping the evolution of all life forms. Organisms have developed structural and physiological systems optimized to function under its constant pull. However, when humanity ventures into space, it is confronted with environments where gravity is either absent or significantly altered. These conditions, including both microgravity and hypergravity encountered in space travel and high‐acceleration manoeuvres, pose unique challenges for human physiology; moreover, activities under controlled gravity offer opportunities of increasing relevance for technological and scientific advancement (Berto et al., 2023).

One of the most sensitive systems affected by gravity variation is the cardiovascular system. In microgravity or low‐gravity environments, the hydrostatic pressure gradient that governs fluid distribution on Earth is eliminated. This leads to a fluid shift from the lower extremities to the upper body, including the thoracic cavity and head. Internal organs expand, reducing compression on the vascular system and altering intrathoracic pressures. As a result, the heart experiences reduced preload resistance, increasing stroke volume as it pumps blood more easily (Norsk, 2020). These seemingly minor haemodynamic changes can, over time, result in cardiac remodelling due to alterations in the delicate balance between atrial–ventricular reciprocal dynamics and ventricular–arterial coupling.

Understanding these physiological adaptations is critical for managing the health of individuals exposed to altered gravity and for leveraging new opportunities in medicine and aerospace. Predicting and analysing even small changes in systemic or pulmonary pressure is a modern medical priority, especially in environments where direct experimentation is limited or practically unfeasible.

In recent decades, computational modelling techniques have become increasingly sophisticated in capturing high‐resolution physiological parameters across various conditions. Of particular value are multiscale models of the cardiovascular system, which can reproduce the physiological behaviour at different levels of complexity. The formulation of these models is guided by a trade‐off between simplicity and fidelity, as increasing the level of detail inherently raises both the system complexity and the volume of required input data. As such, they are composed by elements that range from lumped‐parameter (0D) electrical analogies, to one‐dimensional (1D) schemes that capture flow variations along vessels, to full three‐dimensional (3D) simulations of regions of particular interest, as sketched in Figure 1. Computational models are non‐invasive and capable of simulating physiological responses in scenarios that are otherwise impossible to replicate experimentally – including extreme gravitational conditions.

**Figure 1:**
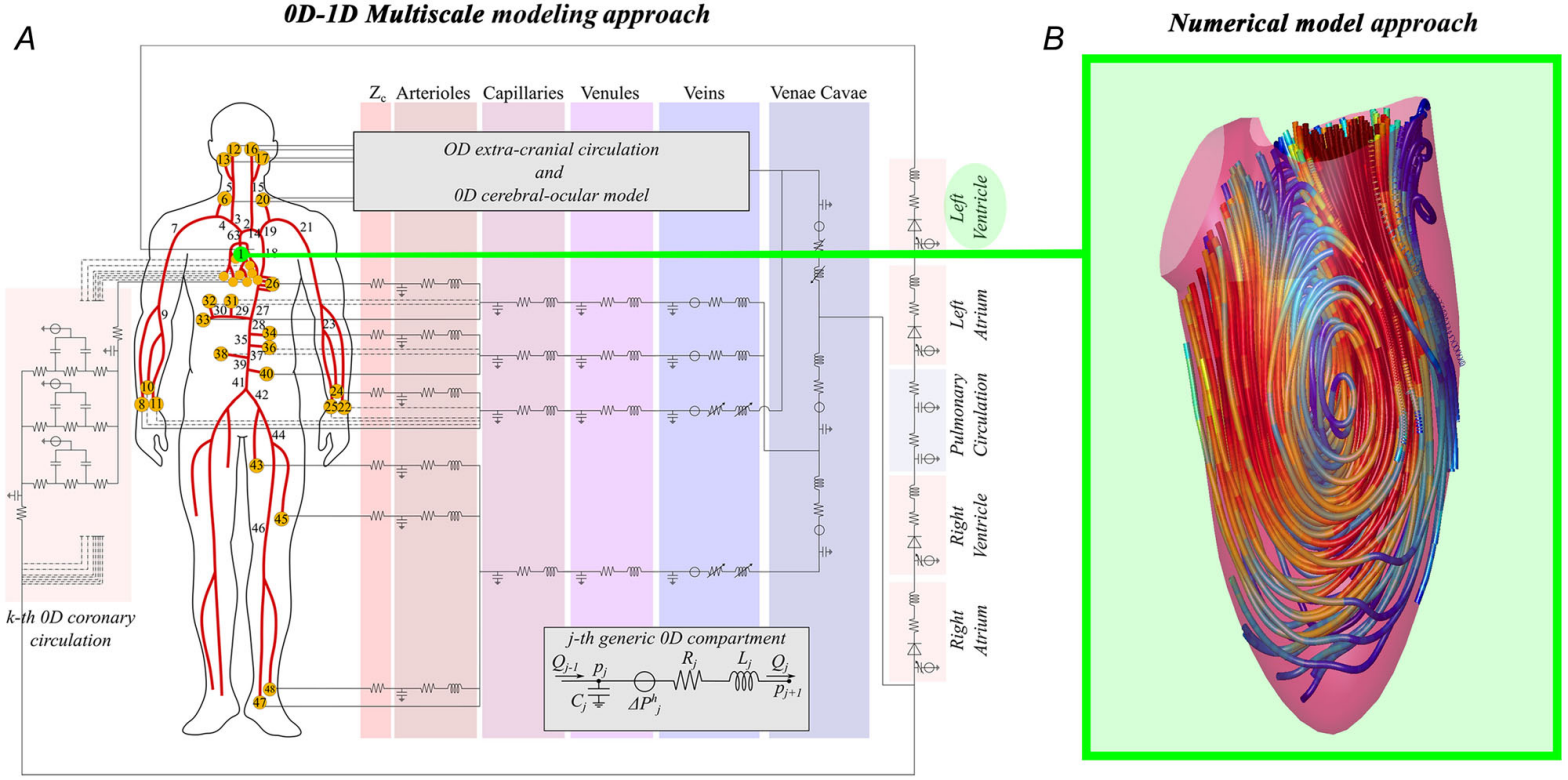
Multiscale modelling: from entire cirulation to local details *A*, a multiscale model of the circulatory system is composed of interconnected elements representing different anatomical regions. These elements typically include lumped (0D) components, analogous to electrical circuits, and linear (1D) segments that simulate flow along vessel tracts. (Adapted from Tripoli et al. 2026.) *B*, advanced models may also incorporate high‐fidelity 3D elements, such as the blood flow dynamics within the left ventricular cavity, enabling detailed investigation of specific physiological phenomena.

In this issue of *The Journal of Physiology*, Tripoli et al. (2026) build on their previous work (Fois et al., 2024) by extending a multiscale model composed of 0D and 1D elements to study the steady‐state cardiovascular response under varying gravity, from 0 *g* (microgravity) to 3 *g* (hypergravity). The study yields important insights; it highlights differential behaviours between the left and right sides of the heart, improved cardiac efficiency in microgravity, and a significant energy supply–demand trade‐off under hypergravity conditions, particularly evident in the right ventricle. Importantly, the model's reliability is supported by comparisons with empirical data from the literature. One of the most relevant findings is the monotonic decrease in end‐diastolic and end‐systolic volumes in both ventricles as gravity increases – an effect attributed to reduced venous return and impaired ventricular filling. Additionally, the study investigates changes in coronary circulation, offering new insights into the gravitational dependence of short‐term haemodynamic responses in both coronary branches.

The study confirms that computational modelling is a valuable complement to experimental research in understanding physiological adaptations to extreme gravity environments. However, challenges remain in translating these findings into clinical practice. Two major directions are worth exploring:

1. Personalized modelling. The current study uses parameters representative of a generic adult male. Future models should explore variations across sex, age and ethnicity, with the ultimate goal of individualized simulation. Such personalized models could significantly enhance risk prediction and countermeasure planning.
2. Integration with high‐fidelity 3D modelling. While the current approach effectively simulates systemic behaviour, further refinement should include detailed 3D modelling of key regions such as the heart chambers. As suggested in Figure 1, integrating myocardial deformation and metrics of intracardiac blood flow patterns, which are known early markers of cardiac remodelling (Vallelonga et al., 2021), could provide deeper insight into long‐term adaptation risks.

In conclusion, the study by Tripoli et al. (2026) marks a significant step toward understanding cardiovascular adaptation to gravitational extremes. Their multiscale computational approach offers a solid foundation for future work aimed at anticipating individual physiological responses, improving countermeasure strategies, and ultimately supporting human health in space exploration and other gravity‐challenging environments.

## Additional information

### Competing interests

The authors have nothing to disclose

### Author contributions

D.C., L.Z. and G.P.: conceived or designed the work; drafted the work or revised it critically for important intellectual content; final approval of the version to be published; agreement to be accountable for all aspects of the work. All authors have approved the final version of the manuscript and agreed to be accountable for all aspects of the work in ensuring that questions related to the accuracy or integrity of any part of the work are appropriately investigated and resolved. All persons designated as authors qualify for authorship, and all those who qualify for authorship are listed.

### Funding

G.P. and L.Z. acknowledge partial support by the Italian Ministry of Education and Research with grant: PRIN 2022AJT27Y, provided through the University of Trieste (CUP J53D23002030006).

## Supporting information


Peer Review History
