# Supplementary material for: From microgravity to hypergravity: Perspectives on circulatory adaptation through multiscale modelling
Source: J Physiol. 2025 Jul 1;604(13):5076–7. doi: 10.1113/JP289417 (PMC13327748; doi:10.1113/JP289417)
Supplement: Supplementary file 1 — Peer Review History [file TJP-604-5076-s001.pdf]

The Journal of Physiology

From Microgravity to Hypergravity: Perspectives on Circulatory Adaptation through Multiscale Modeling

Dario Collia, Luigino Zovatto, and Gianni Pedrizzetti
DOI: 10.1113/JP289417

Corresponding author(s): Gianni Pedrizzetti (gianni.pedrizzetti@dia.units.it)

The following individual(s) involved in review of this submission have agreed to reveal their identity: Stefania Scarsoglio (Referee #1)

Review Timeline:

Submission Date:	13-Jun-2025
Accepted:	17-Jun-2025

Senior Editor: Bjorn Knollmann

Reviewing Editor: Axel Loewe

Transaction Report:

(Note: With the exception of the correction of typographical or spelling errors that could be a source of ambiguity, letters and reports are not edited. Depending on transfer agreements, referee reports obtained elsewhere may or may not be included in this compilation. Referee reports are anonymous unless the Referee chooses to sign their reports.)

Dear Professor Pedrizzetti,

Re: JP-P-2025-289417 "**From Microgravity to Hypergravity: Perspectives on Circulatory Adaptation through Multiscale Modeling**" by Dario Collia, Luigino Zovatto, and Gianni Pedrizzetti

We are pleased to tell you that your paper has been accepted for publication in The Journal of Physiology.

Please see comments below for some small changes that should be made at proof stage.

The last Word (or similar) version of the manuscript provided will be used by the Production Editor to prepare your proof. When this is ready you will receive an email containing a link to Wiley's Online Proofing System. The proof should be thoroughly checked and corrected as promptly as possible.

Authors should note that it is too late at this point to offer corrections prior to proofreading. Major corrections at proof stage, such as changes to figures, will be referred to the Editors for approval before they can be incorporated. Only minor changes, such as to style and consistency, should be made at proof stage. Changes that need to be made after proof stage will usually require a formal correction notice.
All queries at proof stage should be sent to: TJP@wiley.com.

Following copyediting and typesetting the accepted version will be published online.

Yours sincerely,

Bjorn Knollmann
Senior Editor
The Journal of Physiology

IMPORTANT POINTS TO NOTE FOLLOWING YOUR PAPER'S ACCEPTANCE:
If you would like to receive our 'Research Roundup', a monthly newsletter highlighting the cutting-edge research published in The Physiological Society's family of journals (The Journal of Physiology, Experimental Physiology, Physiological Reports, The Journal of Nutritional Physiology, and The Journal of Precision Medicine: Health and Disease), please click this link, fill in your name and email address and select 'Research Roundup':
https://www.physoc.org/journals-and-media/membernews

• TRANSPARENT PEER REVIEW POLICY: To improve the transparency of its peer review process, The Journal of Physiology publishes online as supporting information the peer review history of all articles accepted for publication. Readers will have access to decision letters, including Editors' comments and referee reports, for each version of the manuscript as well as any author responses to peer review comments. Referees can decide whether or not they wish to be named on the peer review history document.

• You can help your research get the attention it deserves! Check out Wiley's free Promotion Guide for best-practice recommendations for promoting your work at: www.wileyauthors.com/eeo/guide. You can learn more about Wiley Editing Services which offers professional video, design, and writing services to create shareable video abstracts, infographics, conference posters, lay summaries, and research news stories for your research at: www.wileyauthors.com/eeo/promotion.

• IMPORTANT NOTICE ABOUT OPEN ACCESS: To assist authors whose funding agencies mandate public access to published research findings sooner than 12 months after publication, The Journal of Physiology allows authors to pay an Open Access (OA) fee to have their papers made freely available immediately on publication.

The Corresponding Author will receive an email from Wiley with details on how to register or log-in to Wiley Authors Services where you will be able to place an order

• You can check if your funder or institution has a Wiley Open Access Account here: https://authorservices.wiley.com/author-resources/Journal-Authors/licensing-and-open-access/open-access/author-compliance-tool.html.

EDITOR COMMENTS

Reviewing Editor:

Thank you for the valuable perspective article.

Senior Editor:

Excellent perspective! Please correct the typos identified by the reviewer in the proof stage.

REFEREE COMMENTS

Referee #1:

The authors clearly and accurately highlight the topic of the focus paper. Just a couple of typos were detected:

Lines 35-36: a trade-off between an simplicity and fidelity -> a trade-off between simplicity and fidelity

Line 61: enhance risk prediction and counter-measure planning -> enhance risk prediction and countermeasure planning
